# Navigating Indigenous Data Sovereignty: A Decolonizing Approach to Understanding Opioid Use Amongst First Nations in Manitoba

**DOI:** 10.23889/ijpds.v9i5.2524

**Published:** 2024-09-18

**Authors:** Wanda Phillips-Beck, Leona Star, Sidney Leggett

**Affiliations:** 1 First Nation Health and Social Secretariat of Manitoba; 2 University of Manitoba

First Nations (FN) and Indigenous Peoples around the world face significant challenges in navigating the complexities of Western research systems, particularly in the context of data sovereignty and research about their people and communities; the same can be said for Western researchers navigating Indigenous research space. We examine the intersection of Western research and FN data to explore opioid use amongst FN in Manitoba and highlight the ethical imperative of utilizing decolonizing frameworks when working with Indigenous data.

Indigenous researchers led a retrospective cohort study linking the Manitoba FN research file to population-level data held at the Manitoba Centre for Health Policy. We centred Indigenous voices, knowledge, protocols, and contextually informed interpretation and fostered meaningful partnerships with academic researchers to ensure we utilized robust statistical methods while prioritizing community-driven questions, needs, and aspirations.

Study period from 2015 to 2019 and included all Manitobans eligible for Manitoba Health Services. We found downward trends in opioid-related use, dispensation, hospitalization, and mortality rates for both FN and all other Manitobans (AOM), but a significant gap was found between FN and AOM for most indicators. Indigenous governance, ownership, engagement, collaboration, reciprocity, and respect for cultural and intellectual property rights are key concepts in ethical research practices that prioritize Indigenous self-determination and data sovereignty.

We underscore the transformative potential of Indigenous-led research and use of decolonizing frameworks in fostering community empowerment to address opioid use in First Nation communities. We upheld the rights of Indigenous peoples to govern their data and undertake their own research.

